# Supplementary material for: Molecular architecture of the assembly of Bacillus spore coat protein GerQ revealed by cryo-EM
Source: Nat Commun. 2024 Sep 16;15:8091. doi: 10.1038/s41467-024-52422-2 (PMC11405398; doi:10.1038/s41467-024-52422-2)
Supplement: Supplementary file 1 — Supplementary Information [file 41467_2024_52422_MOESM1_ESM.pdf]

Title: Molecular architecture of the assembly of *Bacillus* spore coat protein GerQ revealed by cryo-EM

Authors: Yijia Cheng[1,3], Mark A.B. Kreutzberger[2,3], Jianting Han[1], Edward H. Egelman[2*], and Qin Cao[1*]

Affiliations:
[1]Bio-X Institutes, Key Laboratory for the Genetics of Developmental and Neuropsychiatric Disorders, Ministry of Education, Shanghai Jiao Tong University, Shanghai, 200030, China
[2]Department of Biochemistry and Molecular Genetics, University of Virginia School of Medicine, Charlottesville, VA 22903, USA
[3]These authors contribute equally to this work.
[*]Correspondence to: Qin Cao, email: caoqin@sjtu.edu.cn; Edward H. Egelman, email: egelman@virginia.edu

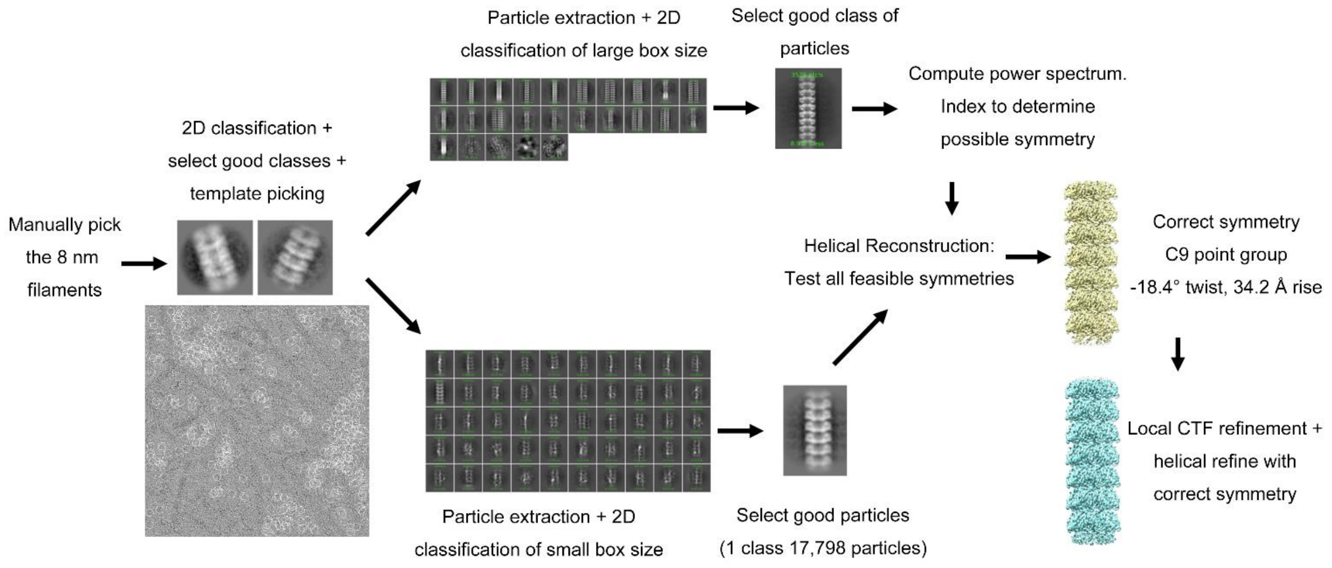

Supplementary Figure 1 Workflow of fibril extraction and cryo-EM structure determination.

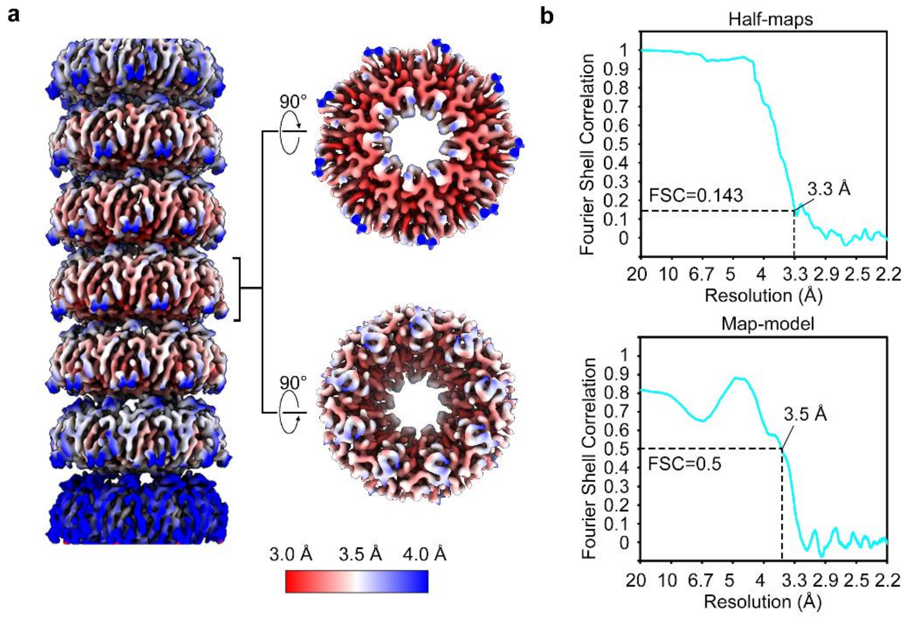

Supplementary Figure 2 Cryo-EM data processing. a, The cryo-EM map of the GerQ filaments colored by local resolution estimate, from red (3.0 Å) to blue (4.0 Å). **b**, FSC curves between two half-maps (top) and the cryo-EM reconstruction and refined atomic model (bottom).

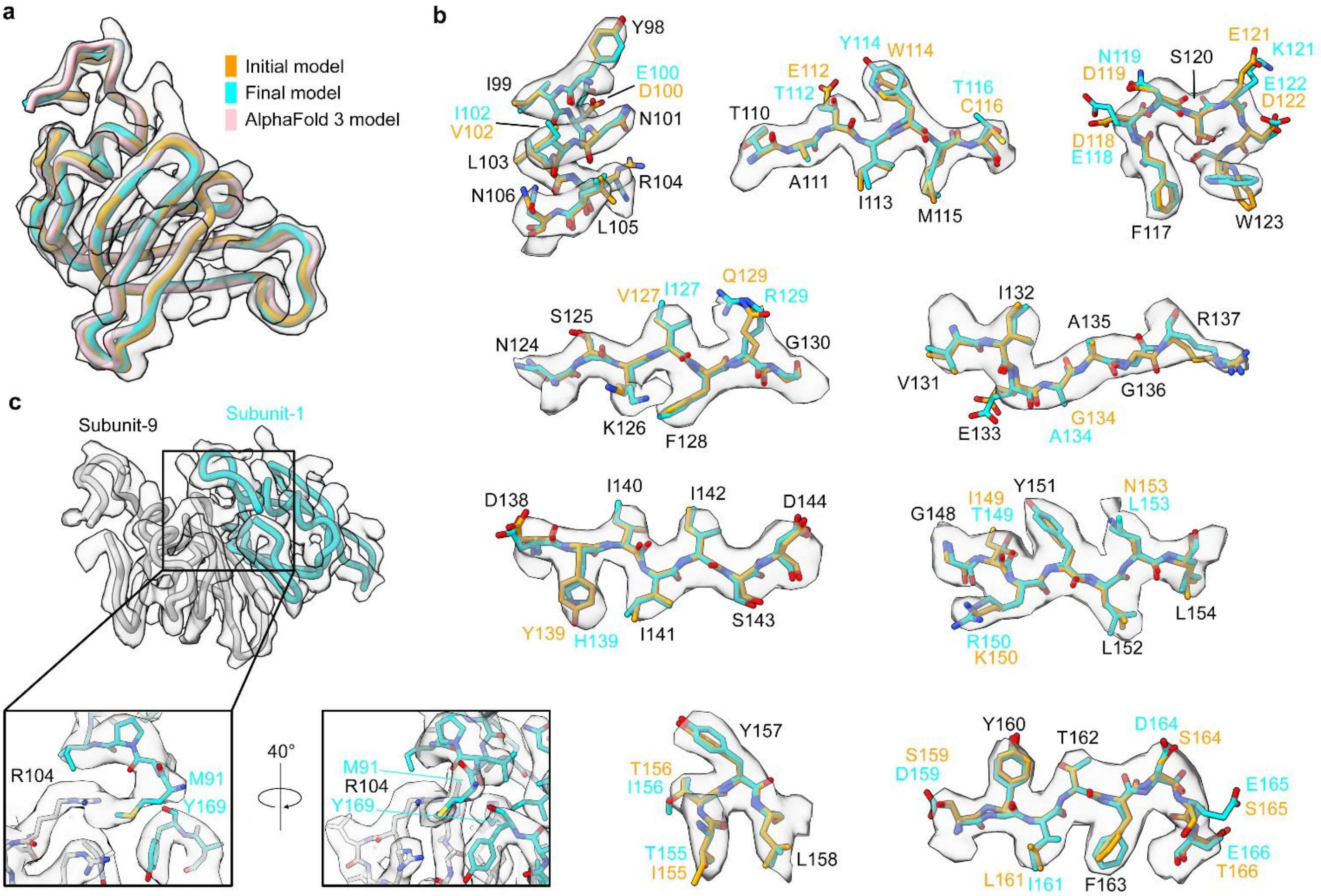

Supplementary Figure 3 Atomic model building. a, Cryo-EM map and superimposition of the model initially generated by ModelAngelo (initial model) and the final model of GerQ, as well as the AlphaFold 3 predicted model. **b**, Detailed comparison between the initial model and the final model. **c**, The connected density between the sidechain of Met91 from one subunit (cyan) and the sidechain of Arg104 from the adjacent subunit (white).

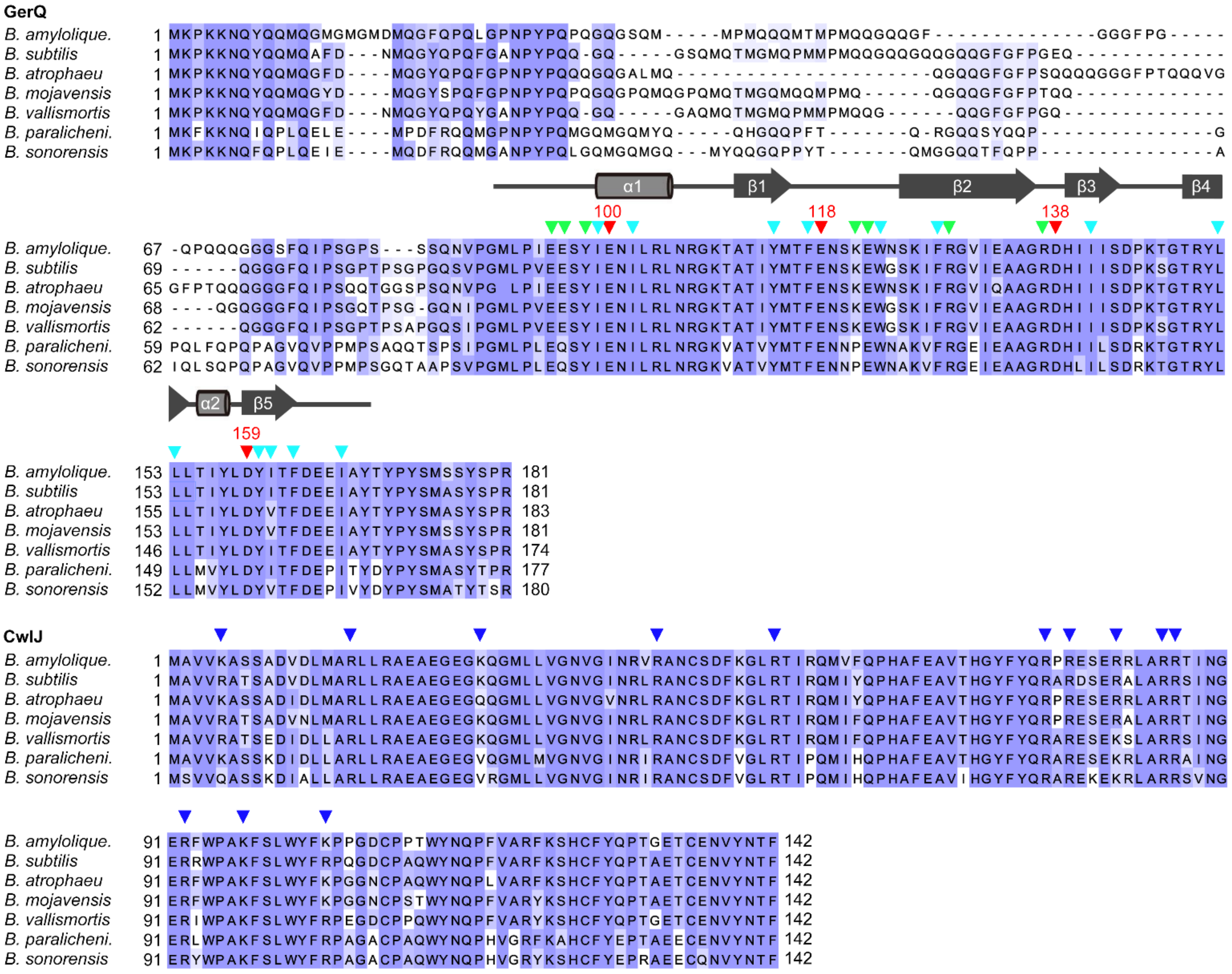

Supplementary Figure 4 Sequence alignment of GerQ and CwlJ in representative *Bacillus* bacteria. Residues involved in the subunit-to-subunit interactions are indicated with cyan arrows, and residues involved in the layer-to-layer interactions are indicated with green arrows. Acid residues present in the inner surface of the bow-like structures of the GerQ filament are indicated with red arrows and labeled with sequence numbers. Basic residues present on the surface of CwlJ that are predicted to interact with GerQ are indicated with blue arrows. The secondary structures of GerQ are aligned with its amino acid sequence.

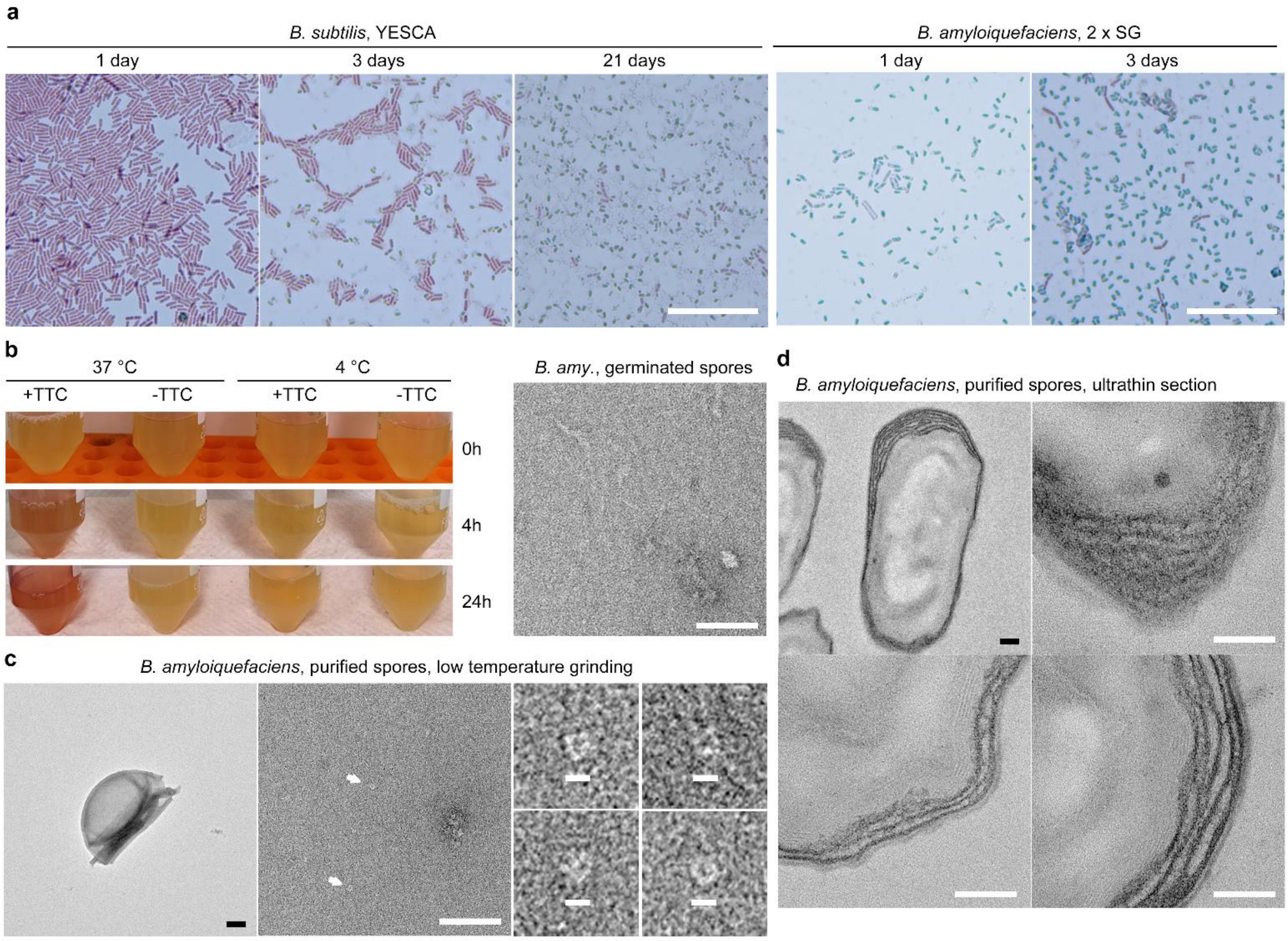

Supplementary Figure 5 Attempts to identify GerQ filaments in other bacteria or under other condition. a, *B. subtilis* were grown on YESCA plates (left panels), and *B. amyloiquefaciens* were grown on 2 × SG plates (right panels), for different durations. Malachite green staining was performed on each sample (top panels, scale bar = 50 µm), with endospores stained green and bacteria stained red. **b**, (left panel) Purified *B. amyloiquefaciens* spores were cultured under varying temperature for different durations and stained with or without 2,3,5-tryphenyltetrazolium chloride (TTC), where germinated spores appear red when stained with TTC. (Right panel) Representative negative stain EM image of germinated spores without additional treatments (scale bar = 100 nm). **c**, Negative stain EM images of low-temperature ground *B. amyloiquefaciens* spores. The ring-like particles observed post-grinding are indicated with white arrows (scale bars in the left and middle panels represent 100 nm, and those in the right panels represent 8 nm). **d**, EM images of ultrathin sections of purified *B. amyloiquefaciens* spores (scale bar = 100 nm). All experiments have been repeated independently for more than three times with similar results.

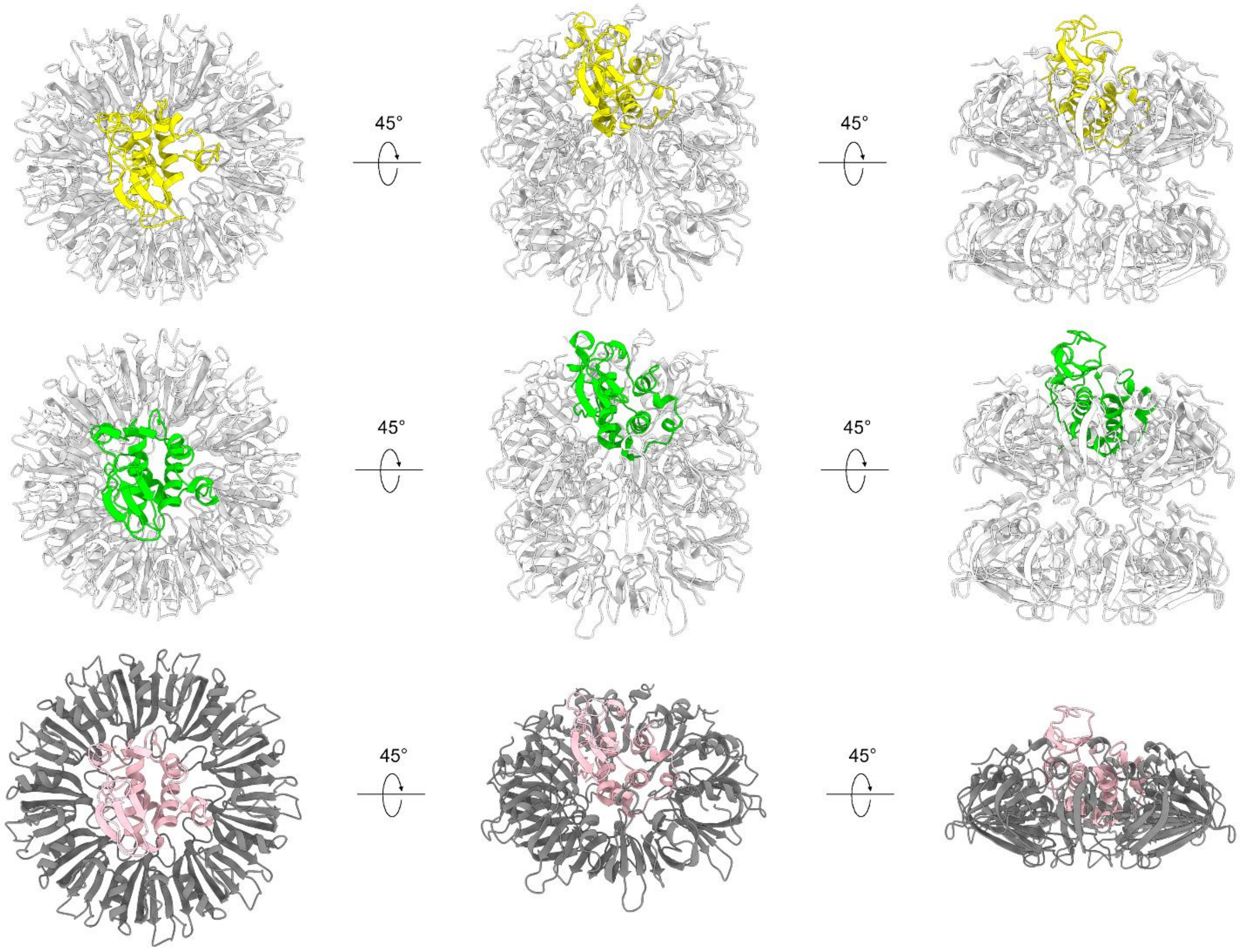

Supplementary Figure 6 Separate views of the macromolecular docking or AlphaFold3 prediction of the GerQ-CwlJ complex. GerQ is colored in white (LightDock and HDock model) or grey (AlphaFold3 model), while CwlJ is colored yellow (LightDock), green (HDock) or pink (AlphaFold3). Both GerQ and CwlJ are displayed as cartoons. For the AlphaFold predicted model, GerQ 91-169 and all residues of CwlJ are displayed.

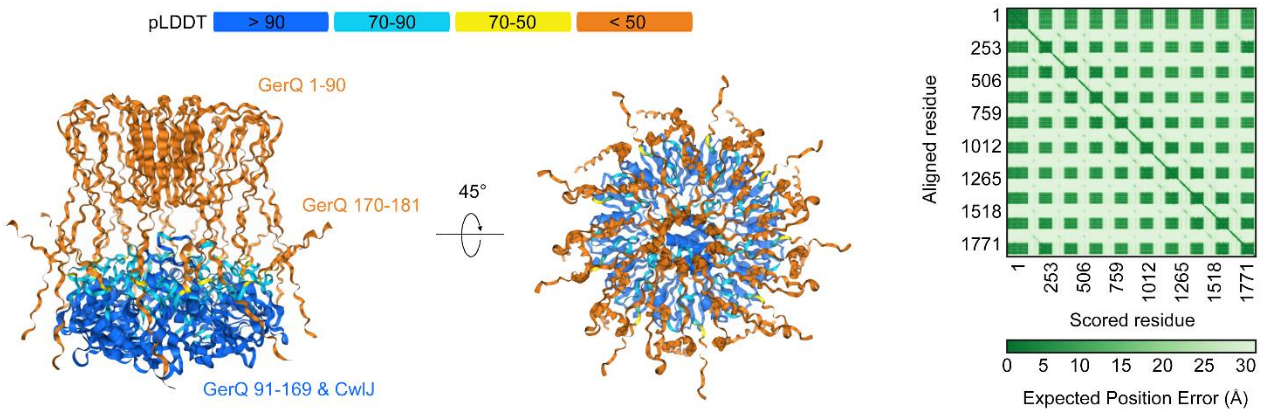

Supplementary Figure 7 Detailed information of the AlphaFold 3 prediction. Predicted model colored by the pLDDT score is shown on the left panels, and the predicted aligned error (PAE) is shown on the right panel. We note that the prediction confidences of the N-terminus and C-terminus region of GerQ (residues 1-90 and 170-181, respectively) are lower than that of the other parts of the GerQ-CwlJ complex. These observations are consistent with our cryo-EM structure, which contains GerQ 91-169 in the filament core.

Supplementary Table 1 Genotyping results

Name	Sequence (5’ to 3’)
Primer, forward	TGGGCTCATATGGTGGAATAG
Primer, reserve	TGGCGTTTATCATCTCTTGG
gerQ	ATGAAACCGAAAAAGAATCAATATCAGCAAATGCAGGGGATGGGGATGGGAATGGATATG<br>CAAGGATTCCAGCCGCAGCTCGGGCCTAATCCTTATCCGCAGCCTCAGGGCCAAGGATCA<br>CAAATGATGCCGATGCAGCAGCAAATGACAATGCCGATGCAGCAAGGCCAGCAGGGCTT<br>CGGCGGGGGGTTCCCCGGCCAGCCGCAGCAGCAGGGCGGCGGCAGCTTTCAAATCCCGT<br>CAGGTCCATCATCGTCACAAAACGTTCCCGGCATGCTGCCGATTGAAGAGTCATACATTG<br>AAAATATTCTGCGGTTGAACCGGGGCAAAACGGCGACGATTTATATGACATTCGAAAACA<br>GCAAAGAATGGAATTCAAAGATTTTCCGCGGTGTGATTGAGGCGGCGGGACGTGACCAT<br>ATCATCATCAGCGATCCGAAAACGGGCACCCGCTATCTGCTTCTGACCATTTACCTTGATT<br>ACATCACATTTGATGAGGAAATCGCTTATACTTATCCGTACTCCATGTCTTCTTATTCTCCG<br>AGATAA
